# The horror of unsafe abortion: case report of a life threatening complication in a 29-year old woman

**DOI:** 10.1186/1754-9493-7-33

**Published:** 2013-10-16

**Authors:** Kaniz Zehra Naqvi, Muhammad Muzzammil Edhi

**Affiliations:** 1 Liaquat National Hospital and Medical Collage, 402, Al jannat plaza, M.A.Jinnah road, Karachi, Pakistan

**Keywords:** Unsafe abortion, Bowel injury, Surgical complication, Patient safety

## Abstract

**Background:**

Every year 42 million women with unintended pregnancies choose abortion, and fifty percent of these procedures, 20 million are unsafe. An unsafe abortion is defined as a procedure for terminating an unintended pregnancy carried out either by person lacking the necessary skills or in an environment that does not conform to minimal medical standards or both.

Pakistan is the one of the six countries where more than 50% of the world’s all maternal deaths occur. It is estimated that 890,000 induced abortions are performed annually in Pakistan, and estimate an annual abortion rate of 29 per 1000 women aged 15-49.

**Case presentation:**

Here we present a case report of a 29-year old woman who underwent an unsafe abortion for unintended pregnancy resulting in uterine perforation. The unskilled provider pulled out her bowel through vagina after perforating the uterus, as a result she lost major portion of her small intestine resulting in short bowel syndrome.

**Conclusion:**

The law of Pakistan only allows abortion during early stages of pregnancy for purpose of saving the life of a mother but does not cater for cases of rape, incest and fetal abnormalities or social reasons.

Only legalization of abortion is not sufficient, preventing unintended pregnancy should be the priority of all the nations and for this reason contraception should be widely accessible.

Practitioners need to become better trained in safer abortion methods and be to able transfer the patient to health facility when complications occur.

## Background

Pakistan is the one of the six countries where more than 50% of the world’s all maternal deaths occur [1].

It is estimated that 890,000 induced abortions are performed annually in Pakistan, and estimate an annual abortion rate of 29 per 1000 women aged 15-49 [2].

According to World Health Organization, every 8 minutes a women in a developing nations will die of complications arising from an unsafe abortion [3]. The fifth United Nations Millennium Development Goal recommends 75% reduction in maternal mortality by 2015. WHO deems unsafe abortion one of easiest preventable causes of maternal mortality and a public health issue.

Throughout Europe, except for Ireland and Poland, abortion is broadly legal, widely available and safe. Even United States legalized abortion nationwide and this is because of the realization that restrictive policies were instead of ending abortion were putting pressure on public health especially on those who could not afford to pay for safe abortion. Today, 60% of the world’s 1.55 billion women of reproductive age(15-44) live in countries where abortion is legal, the remaining 40% live where abortion is highly restricted, virtually all of them in developing countries [4]. Data suggests that even as the overall abortion rate has declined, the proportion of unsafe abortions is on the rise, especially in the developing nations [5]. It is clear that in those countries where contraceptive use increased the most, abortion rate dropped significantly but in countries like Pakistan which has 25% unmet need of contraception the incidence of unsafe abortion is still high 29 per 1000 women aged 15-49 [2].

Approximately 1 in 10 pregnancies end in an unsafe abortion, giving a ratio of 1 unsafe abortion to about 7 live births [6]. Approximately eighty million more women per year suffer post abortion complications that can lead to short or long term consequences [4].

Highest incidence of unsafe abortion takes place in Latin America, Africa and South East Asia [3]. According to Pakistan demographic survey 2006–7 with total fertility rate at 4.1%, stagnant contraception prevalence rate 29.6% and high 25% unmet need for contraception, and 1 out of every 4 birth unwanted, prospects of achieving MDG 4and 5 by 2015 look bleak [7].

It should not therefore come as a surprise that unwanted pregnancies are the leading cause of induced abortion in Pakistan [8].

40% of these abortions are performed by unskilled workers in back street clinics.

It is seen that in countries with restrictive laws, the women who are determined to end an unwanted pregnancy will seek out clandestine means. In Pakistan where average earnings of a person are less than $2 per day and fee for doctor assisted abortion is around $50-104, the services provided by untrained persons thrive. The shaming, blaming and the judgemental or punitive attitude of the staff are another factor which prevents these females from seeking post abortion medical care. So changing the laws is no guarantee that unsafe abortion will not take place. In Zambia a study findings revealed, high ratio of induced abortion mortality and more than half of those deaths were of schoolgirls. Although abortion is legal in Zambia on social and medical grounds but most females choose illegal abortion because of being expelled from school, unwillingness to reveal relationship, to protect the health of their previous baby [9].

The main causes of death or morbidity from unsafe abortion is due to haemorrhage, sepsis, genital trauma and bowel injury. Here we are presenting a case report of unsafe abortion in a young woman which resulted not only in unrecognized perforation of uterus, but also the removal of a significant portion of her small intestines via the uterine perforation and introitus causing severely shortened intestines and infection. The procedure was performed by an unskilled worker in one of the back street clinic of the city.

## Case report

At 9 pm a ‘29-year old female’ Para 0 ^+0^ was admitted via Accident and Emergency department of our hospital complaining of severe abdominal pain starting earlier in the afternoon. She reported recent attempts at termination of a 10 weeks unplanned and undesired pregnancy at an outside clinic. According to the patient about three to four weeks earlier as a part of workup done for fever revealed pregnancy of about 10 weeks duration. She took some abortificient to abort this unintended pregnancy. She developed bleeding per vagina following that, for which she had uterine evacuation at some small clinic. After that she came home, but next day she started to bleed heavily per vagina, so she went back to the same place and was prescribed tablet misoprostol twice daily. According to her she took this tablet for 1 week but as she continued to bleed so she again visited the same clinic and second uterine evacuation was performed on her. After 2 or 3 days she returned to the same clinic because her bleeding had not yet subsided. A third attempt on uterine evacuation was made but this time there was lots of pain which was unbearable, so the person attempting the evacuation gave her some intravenous sedation and completed her job. After returning home, the patient almost collapsed due to severe pain, so her family brought her to hospital.

On presentation to our hospital she was conscious, pale and in obvious discomfort, her BP was 115/77 mm Hg, pulse 99/min, temperature was 99.2°F. Abdominal examination revealed generalized tenderness and guarding all over the abdomen. Bowel sounds were absent. On per vagina examination, there was no active bleeding but vagina was hot, uterus was about 10–12 weeks size, mobile and cervical os was closed. Her blood investigation showed Hb 7 gm/dl, white cell count 9.6 × 10^9^/l, platelets were 278 × 10^9^/l. urea, creatinine and electrolytes were all within normal limits. Ultrasound pelvis showed fluid with echoes in pelvis, an empty uterus and normal looking ovaries. Suspecting uterine/bowel injury we also asked for x-ray abdomen both erect and supine and it showed gas under the diaphragm. A clinical diagnosis of uterine perforation leading to bowel injury was made and laparotomy planned after resuscitation of patient.

**Figure 1:**
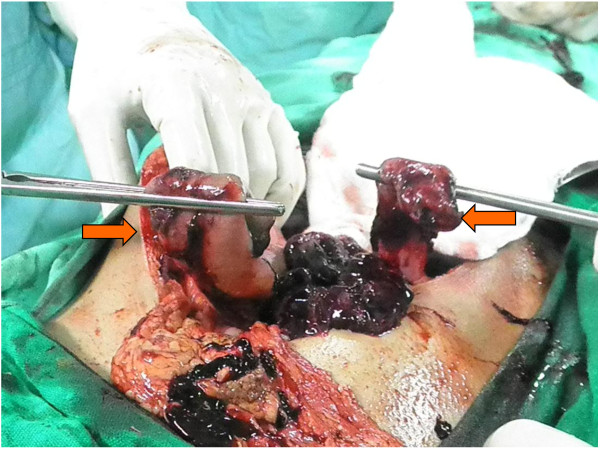
Cut ends of small bowel.

During the exploratory laparotomy, hemoperitoneum of about 500–800 ml was noted, additionally two separate segments of small bowel were identified lying at a distance from each other and in between mesentery was all bruised and necrosed (Figure 1).

**Figure 2:**
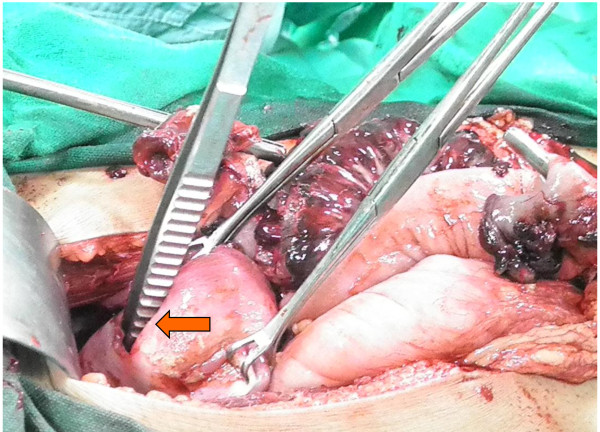
Perforation in anterior wall of uterus.

When the bowel was run, we found that only about one and half feet of small bowel from duodeno-jejunal flexure and about 6 inches from ilieo-caecal junction intact, rest of the small bowel was missing completely. It transpired that while doing the evacuation the person had removed the whole of small gut except for those two small pieces. We also found a 2.5 cm perforation in the anterior wall of uterus close to cervical canal (Figure 2).

After resecting the nonviable portion of intestines an end to end anastomosis was performed. Primary repair of uterine perforation was done. Abdominal cavity washed and closed leaving a drain behind. Post operatively she was kept in high dependency unit. 3 units of packed red cells were transfused, broad spectrum antibiotics and intravenous fluids were given. She responded well to the treatment, on third post-operative day the intra-abdomen drain was removed and she was discharged on tenth post-operative day.

At the time of discharge the patient and her family was counselled regarding the implications of losing a major portion of her small bowel. They were told that she will suffer from repeated bouts of diarrhoea which may cause dehydration and malnutrition. Advice regarding small frequent meals, fluid in the form of ORS, nutritional supplements and medication to control diarrhoea was given. She was also referred to psychologist for support and therapy.

She was admitted twice through accident and emergency for treatment of dehydration because of diarrhoea. She has been under regular follow up and though has lost weight but her diarrhoea has improved.

Psychological support in the form of counselling of both the family and the patient was carried out but since there are no established support groups so whatever was done was on individual basis.

## Discussion

Uterine perforation and bowel injuries are the major complications after unsafe abortion. The reason for these complications is that most abortions are done by untrained personals i.e. unskilled workers in very unhygienic conditions [10,11]. The same happened with this unfortunate woman, the person doing the evacuation did not recognise that she had perforated the uterus and what she was pulling out was intestines and as a result this woman ended up with only one and half to two feet of small intestine. In one study 11.2% had bowel injury and most of the abortions were performed by unskilled workers [12]. In another study done at Khyber medical college and hospital in Peshawar Pakistan the incidence of gut injury after induced abortion was about 42% [13]. Despite the adverse outcome of abortions, the low socio-economic status of these women compels them to resort to abortion rather than practicing contraception as it entails a ‘one time’ cost compared to recurrent cost of buying contraception [8]. These unqualified providers are easily accessible to the clients in countries such as Pakistan.

Even safe abortions in the developing countries are still risky because it depends on the health facility, the training of the provider and the gestational age of the fetus. With unsafe abortion the risk of maternal morbidity and mortality depends on method of abortion and the willingness of the women to seek post abortion care [14].

Data on nonfatal long term health consequences are poor, but those documented are infertility, stool or urinary incontinence due to bowel or bladder injury and bowel resection along with psychological trauma.

There is a relationship between unsafe abortion and restrictive abortion laws. The median rate of unsafe abortions in the 82 countries with the most restrictive abortion laws is up to 23 of 1000 women compared with 2 of 1000 in nations that allow abortion [15].

Less restrictive abortion laws do not appear to increase the abortion rate overall. The world’s lowest rate is in Europe, where abortion is legal and easily available because the contraception use is high. Compared to Latin America, Africa and south east Asia where abortion laws are more restrictive and contraception use is low the rates ranges from mid 20 s to 39 per 1000 women [16].

In developing countries, two third of unintended pregnancies occur in women who are not using any contraception.

Complications due to unsafe abortion account for an estimated 13% of maternal deaths world over or 70,000 deaths per year [17].

Unsafe abortion is a significant problem both medical and social worldwide. It is seen that in developing countries most unsafe abortions are carried out by untrained persons resulting in high morbidity and mortality [18].

## Conclusion

To reduce the morbidity and mortality associated with unsafe abortions, intensive dissemination of information and commitment at all levels is required. Use of various contraceptive methods should be promoted in order to prevent unintended pregnancies. Governments and non government organizations should find ways and means to overcome cultural and social misconceptions which restrict women from receiving health care.

Regular training courses for traditional birth attendants, nurses and doctors under the supervision of expert obstetrician should be carried out. All those facilities which provide such services should have appropriate equipment and trained staff and the service is provided at a reasonable cost. Post abortion family planning counselling should be the part of the service.

There is evidence that liberalizing abortion laws results in reduction in abortion related morbidities and mortalities but here the role of socio-political and religious organization comes into play.

By preventing 5 million abortions related complications and deaths worldwide we can save 220,000 children from becoming motherless.

## Consent

Written informed consent was obtained from the patient for publication of this Case report and accompanying images. A copy of the written consent is available for review by the Editor-in-Chief of this journal.

## Competing interest

The authors declare that they have no competing interests.

## Authors’ contributions

MME did manuscript drafting and KZN did critically review the manuscript. Both authors approved the final document of manuscript.
